# The Cysteine Desulfurase IscS Is a Significant Target of 2-Aminoacrylate Damage in Pseudomonas aeruginosa

**DOI:** 10.1128/mbio.01071-22

**Published:** 2022-06-02

**Authors:** Ronnie L. Fulton, Jessica Irons, Diana M. Downs

**Affiliations:** a Department of Microbiology, University of Georgia, Athens, Georgia, USA; University of Illinois at Urbana Champaign; University of Hawaii at Manoa

**Keywords:** RidA, PA5339, IscS, 2-aminoacrylate, aminoacrylate stress

## Abstract

Pseudomonas aeruginosa encodes eight members of the Rid protein superfamily. PA5339, a member of the RidA subfamily, is required for full growth and motility of P. aeruginosa. Our understanding of RidA integration into the metabolic network of P. aeruginosa is at an early stage, with analyses largely guided by the well-established RidA paradigm in Salmonella enterica. A P. aeruginosa strain lacking RidA has a growth and motility defect in a minimal glucose medium, both of which are exacerbated by exogenous serine. All described *ridA* mutant phenotypes are rescued by supplementation with isoleucine, indicating the primary generator of the reactive metabolite 2-aminoacrylate (2AA) in *ridA* mutants is a threonine/serine dehydratase. However, the critical (i.e., phenotype determining) targets of 2AA leading to growth and motility defects in P. aeruginosa remained undefined. This study was initiated to probe the effects of 2AA stress on the metabolic network of P. aeruginosa by defining the target(s) of 2AA that contribute to physiological defects of a *ridA* mutant. Suppressor mutations that restored growth to a P. aeruginosa *ridA* mutant were isolated, including an allele of *iscS* (encoding cysteine desulfurase). Damage to IscS was identified as a significant cause of growth defects of P. aeruginosa during enamine stress. A suppressing allele encoded an IscS variant that was less sensitive to damage by 2AA, resulting in a novel mechanism of phenotypic suppression of a *ridA* mutant.

## INTRODUCTION

The Rid (YjgF/YER057c/UK114) protein superfamily is divided into nine subfamilies (RidA, Rid1-7, RutC-like) based on a phylogenetic grouping by the NCBI Conserved Domain Database (cd00448: YjgF_YER057c_UK114_family) (1). Members of the RidA (reactive intermediate deaminase A) subfamily are present in all domains of life, while Rid1-7 subfamilies exist only in prokaryotic genomes. RidA homologs from mammals, plants, yeast, and bacteria have been noted in the literature for the past 2 decades (2). After identification of the enamine/imine deaminase activity of RidA from Salmonella enterica (3, 4), similar activity was demonstrated for the human (UK114), goat (UK114), cucumber (ChrD), Pyrococcus furiosus (PF0668), Bacillus subtilis (YabJ), Pseudomonas aeruginosa (PA5339), Campylobacter jejuni (Cj1388), Saccharomyces cerevisiae (Mmf1p), Yersinia pestis (Y3551), and dust mite (Der F34) homologs *in vitro* (3, 5–10).

**FIG 1:**
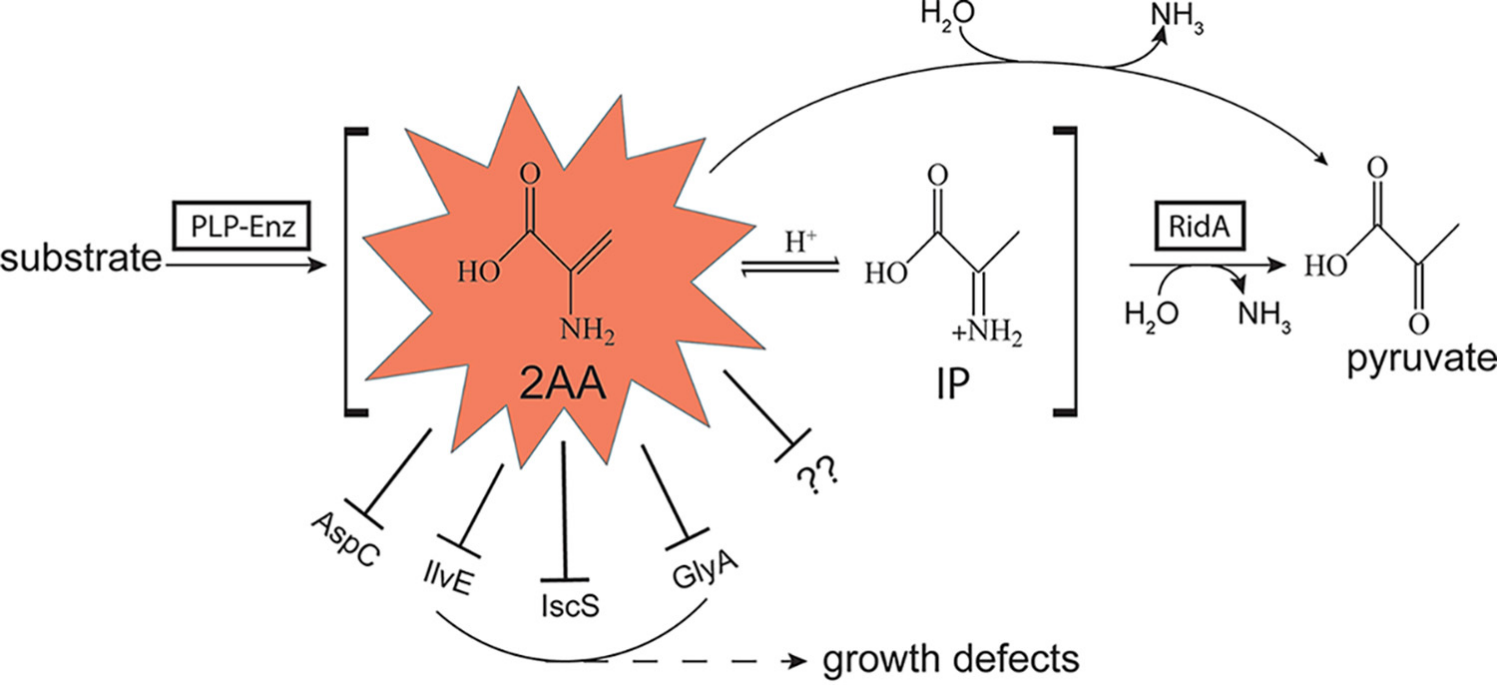
General RidA paradigm. Some PLP-dependent enzymes (PLP-Enz) generate an enamine intermediate (2-aminoacrylate [2AA]), which can tautomerize to the imine 2-iminopropionate (IP). 2AA is deaminated to pyruvate by RidA, or spontaneously by free water. In the absence of RidA, spontaneous deamination by water in the cellular milieu is not sufficient and 2AA persists. If allowed to persist in the cell, 2AA can irreversibly damage multiple PLP-dependent enzymes, as schematically represented. Depending on the metabolic architecture of the organism, one or more of these damaged enzymes will generate nutritional and/or growth phenotypes in *ridA* mutants. In S. enterica, GlyA is the most significant enzyme damaged in the sense that bypassing this step with exogenous glycine reverses the majority of the growth defects. In Pseudomonas aeruginosa, damage to IscS was the primary driver of growth consequences of a *ridA* mutant. Question marks represent the additional enzyme targets that are not yet identified. Abbreviations: AspC, aspartate transaminase (EC 2.6.1.1); IlvE, transaminase B (EC 2.6.1.42); IscS, cysteine desulfurase (EC 2.8.1.7); GlyA, serine hydroxymethyltransferase (EC 2.1.2.1).

The RidA paradigm of 2-aminoacrylate (2AA) stress was elucidated primarily by biochemical and genetic studies in S. enterica (Fig. 1). Generation of 2AA, and its subsequent release from the active site, has been shown for three enzymes in S. enterica: threonine/serine dehydratase (IlvA, EC 4.3.1.19), cysteine desulfhydrase (CdsH, EC 4.4.1.15), and diaminopropionate ammonia-lyase (DapL, EC 4.3.1.15) (3, 11, 12), which use serine, cysteine, or diaminopropionate, respectively, as the substrates. 2AA is generated primarily by the serine/threonine dehydratase, IlvA, in S. enterica. In the absence of RidA, 2AA persists and damages pyridoxal 5′-phosphate (PLP)-dependent enzymes, often causing detectable phenotypic consequences. To date, the PLP-dependent enzymes shown to be inactivated by 2AA *in vivo* include branched-chain amino acid aminotransferase (IlvE, EC 2.6.1.42), alanine racemases (Alr/DadX, EC 5.1.1.1), serine hydroxymethyltransferase (GlyA, EC 2.1.2.1), aspartate aminotransferase (ApsC, EC 2.6.1.1), and aminolevulinic acid synthase (Hem1p, EC 2.3.1.37) (4, 13–17). A study of the consequences of 2AA stress in the metabolic network of various organisms has uncovered similarities and differences in the generators and targets of 2AA when comparing S. enterica to Escherichia coli, P. aeruginosa, C. jejuni, and S. cerevisiae (6–8, 16, 17).

P. aeruginosa encodes nine members of the Rid superfamily with two (PA3123 and PA5339) that belong to the RidA subfamily (7). Despite encoding multiple Rid family members, only a mutation in *PA5339* led to metabolic defects characteristic of a *ridA* mutant, leading to its designation as *ridA* (7). P. aeruginosa *ridA* mutants have a defect in both growth and motility in a minimal glucose medium that is corrected by exogenous isoleucine. P. aeruginosa *ridA* mutants were sensitive to lower concentrations of serine than S. enterica *ridA* mutants, and only slightly sensitive to cysteine. These data suggest that the generation of 2AA in P. aeruginosa proceeds largely through the two serine/threonine dehydratases encoded by IlvA paralogs, PA0331 and PA1326. The targets of 2AA in P. aeruginosa that are responsible for growth defects differ from those in S. enterica and E. coli. This conclusion is based on observations that the growth of P. aeruginosa *ridA* mutants is improved by the addition of isoleucine or threonine, but not by exogenous glycine or aspartate (7). The latter two supplements restore growth to *ridA* mutants of S. enterica and E. coli, respectively (16, 18). The differences between the phenotypes of a *ridA* mutant in S. enterica and P. aeruginosa underscore the differences in metabolic networks between organisms and how these differences can influence the phenotypic output of the system.

Motility defects have been observed in *ridA* mutants of S. enterica, P. aeruginosa, and C. jejuni, suggesting 2AA damage has some broadly conserved consequences despite the distinct metabolic networks of each organism (7, 8, 19). The cause of the motility defect resulting from the lack of RidA, and whether it is a direct or indirect consequence of 2AA damage, is not known for any organism thus far. The conserved deaminase activity of RidA proteins from all domains of life suggests the consequences of *ridA* inactivation can be attributed to the accumulation of 2AA and the resulting damage to PLP-dependent enzymes. Expansion of the RidA paradigm to multiple organisms provides an opportunity to gain insight into the unique metabolic network of each organism and gain an understanding of the broader effects of RidA activity and 2AA stress on metabolism.

Previous studies identified the functional RidA in P. aeruginosa but the integration of this protein, and broader consequences of 2AA accumulation in this organism, have not been described. Classical genetic suppressor analysis was used herein to investigate the consequences of enamine stress in P. aeruginosa and the role of RidA in ameliorating it. Of the three suppressors identified, an allele of *iscS* (PA3814; encoding a cysteine desulfurase; EC 2.8.1.7) provided new insights into the consequences of 2AA damage and identified IscS as a significant target of 2AA in P. aeruginosa.

## RESULTS

### Spontaneous mutations suppressed phenotypes of a P. aeruginosa *ridA* mutant.

P. aeruginosa *ridA* mutants have a significant reduction in motility and a severe growth defect in the presence of serine compared to wild-type (7). While both phenotypic defects are the result of accumulated 2AA (7), the target(s) of 2AA responsible for either phenotype has not been defined. Spontaneously arising suppressor mutations that restored growth of the *ridA* mutant on a minimal glucose medium with serine (0.5 mM) were isolated. Colonies appeared after incubation for 72 h and five independent colonies were further characterized. Separately, mutations were selected for the ability to overcome the motility defect. After 5 days of incubation in motility agar, outgrowths from the motility halo were observed on ~85% of plates. Single colonies from these outgrowths were isolated. Ultimately, two mutants selected in the presence of serine and a single mutant with restored motility were chosen for further characterization. Two of the three genomes contained high probability SNPs, while the third contained an in-frame deletion. Mutations were identified in *PA3814* (*iscS*), *PA1010* (*dapA*) and *PA1559*, respectively. In each case, Sanger sequencing confirmed the lesion identified by whole-genome analysis was present in the relevant strain. The three strains were phenotypically characterized to confirm suppression of the *ridA* mutant phenotype(s) (Table 1).

**TABLE 1:** Strains, plasmids, and primers

Plasmid name, strain ID, or primer	Description, genotype^*a*^, or sequence	Drug resistance	Source
pCV1	pCV1 – empty vector	Ap^R^	47
pDM1439	pCV1 – S. enterica RidA	Ap^R^	Laboratory collection
pDM1636	pCV1 – P. aeruginosa IscS	Ap^R^	This study
pDM1637	pCV1 – P. aeruginosa IscS^Q183P^	Ap^R^	This study
pDM1684	pTEV18 – P. aeruginosa IscS	Ap^R^	This study
pDM1685	pTEV18 – P. aeruginosa IscS^Q183P^	Ap^R^	This study
P. aeruginosa			
DMPA4	MPAO1 wild-type (Manoil laboratory)		40
DMPA5	*ridA*-F05::ISphoA/hah *dapA*_(A321G, A322G)_		This study
DMPA7	r*idA*-F05::ISphoA/hah		40
DMPA13	*ridA*-F05::ISphoA/hah *PA1559*_(ΔTCA188)_		This study
DMPA14	*ridA*-F05::IsphoA/hah *iscS*_(A548C)_		This study
S. enterica			
DM5419	*iscR1*::MudJ^*b*^ (25)		Laboratory collection
DM12920	*ridA1*::Tn*10d* (Tc)^*b*^		Laboratory collection
DM13509	*aadA*::araCpBADT7 (SM300A1→His+) (45))		Laboratory collection
DM14846	*ridA1*::Tn*10d* (Tc)/pDM1439		This study
DM14847	*ridA1*::Tn*10d* (Tc)/pCV1		This study
DM17050	*aadA*::araCpBADT7 *ridA1*::Tn*10*(d)		Laboratory collection
DM17142	*ridA1*::Tn*10d* (Tc)/pDM1636		This study
DM17143	*ridA1*::Tn*10d* (Tc)/pDM1637		This study
DM17174	*iscR1*::MudJ/pDM1636		This study
DM17175	*iscR1*::MudJ/pDM1637		This study
DM17194	*iscR1*::MudJ/pCV1		This study
DM17392	*aadA*::araCpBADT7/pDM1684		This study
DM17393	*aadA*::araCpBADT7/pDM1685		This study
DM17394	*aadA*::araCpBADT7 *ridA1*::Tn*10*(d)/pDM1684		This study
DM17395	*aadA*::araCpBADT7 *ridA1*::Tn*10*(d)/pDM1685		This study
PAiscS pBAD For	NNGCTCTTCNTTCATGAAATTGCCGATCTACCTC		
PAiscS pBAD Rev	NNGCTCTTCNTTATCAGTGCCCTGCCATTC		
pae-TEV18-iscS-F	cgaagagcgctcttcttaagATGAAATTGCCGATCTACCTCG		
pae-TEV18-iscS-R	ggccgcggatcccgggagctTCAGTGCGCCTGCCATTC		

a Mutant alleles in P. aeruginosa were designated by the respective nucleotide changes as a subscript.

b Tn10d(Tc) was the transposition-defective mini-Tn10 (Tn10Δ16Δ17) described by Way et al. (48). MudJ refers to the MudJ1734 transposon (49).

### Diverse suppressor mutations restored growth with serine.

**FIG 2:**
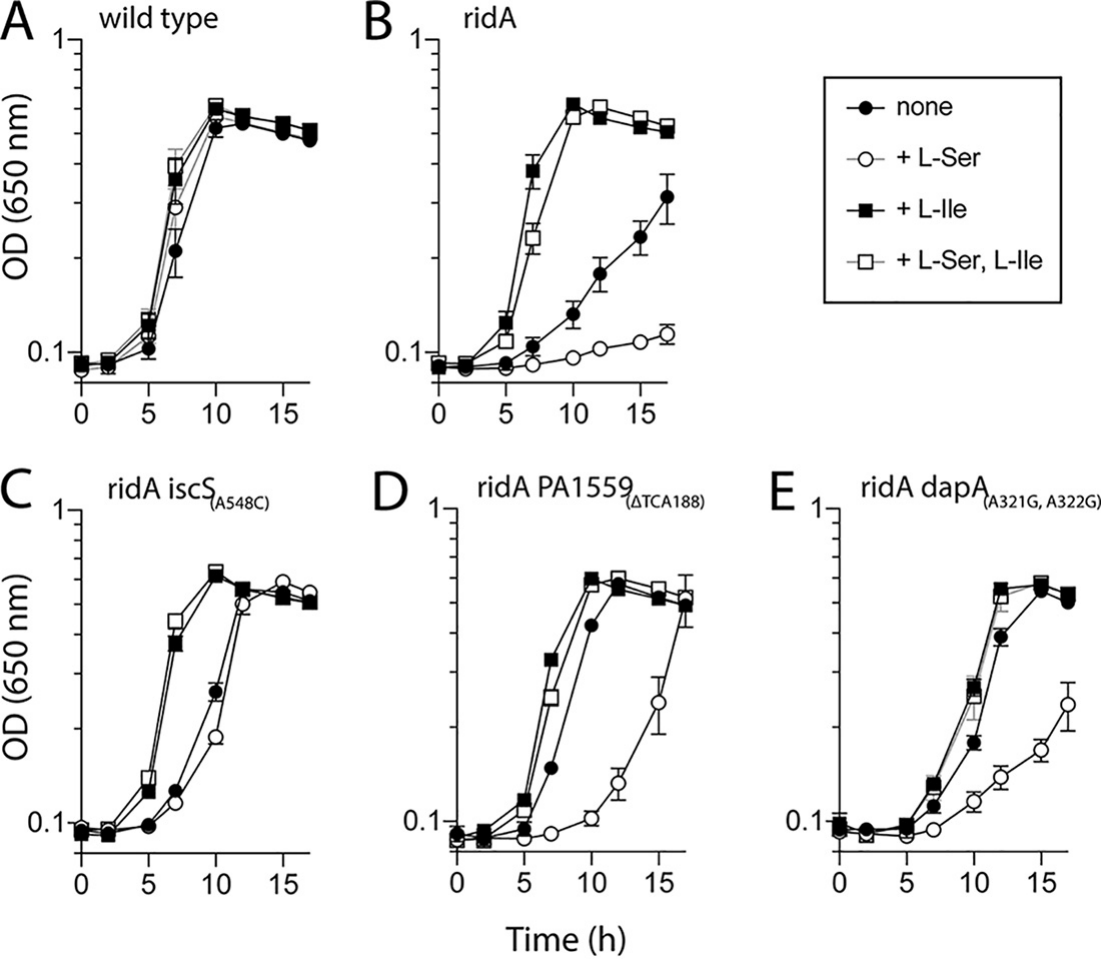
Suppressor mutations alleviate the growth defect of a P. aeruginosa *ridA* mutant. Bacterial growth was measured as the change in optical density over time when strains were inoculated into minimal glucose (11 mM) medium with no supplements or with l-serine (0.5 mM), l-isoleucine (1 mM), or both l-serine and l-isoleucine added as indicated. Growth patterns of wild-type P. aeruginosa (A), P. aeruginosa *ridA* (B), and *ridA* mutants with spontaneous suppressor mutations, *iscS*_(A548C)_ (C), *PA1559*_(ΔTCA188)_ (D), and *dapA*_(A321G, A322G)_ (E) are shown. Error bars represent the standard deviation between three independent biological replicates.

Growth of P. aeruginosa *ridA*, and suppressor mutants DMPA14 (*iscS*), DMPA13 (*PA1559*), and DMPA5 (*dapA*) in liquid glucose media with serine (0.5 mM) and/or isoleucine (1 mM) is shown in Fig. 2. Compared to wild-type, the *ridA* mutant strain had a significant growth defect in minimal medium and was unable to grow in the presence of serine, while isoleucine supplementation restored full growth in both media (Fig. 2A and B). The phenotypic profile of the suppressors separated them into two classes. The lesions in *dapA* and *PA1559* restored full growth in minimal medium and significantly increased growth in the presence of serine (Fig. 2D and E). In contrast, the lesion in *iscS* marginally increased growth on a minimal medium and eliminated the impact of serine on growth (Fig. 2C). Significantly, growth of the *iscS* suppressor mutant was not restored to wild-type levels in either medium. In total, nutritional analyses showed that (i) each of the P. aeruginosa *ridA* suppressor mutants was less sensitive to serine, (ii) isoleucine restored wild-type growth under all conditions tested, and (iii) the growth pattern allowed by the *iscS* mutation was distinct among the suppressors.

### Suppressor mutations increased motility.

Each of the three suppressor mutants improved motility in minimal glucose motility agar (Table 2). On average, a *ridA* mutant was ~50% as motile as the wild-type. All suppressor mutants were significantly more motile than the parental *ridA* mutant. The *iscS* suppressor mutation was less efficient at restoring motility than the other two mutations. As observed previously (7), isoleucine restored full motility (Table S1). It was formally possible that differences in motility reflected different growth rates of the mutants. This possibility was minimized by assessing motility in the presence of thiamine, which restored growth but not motility (Table 2).

**TABLE 2:** Spontaneous mutations restored motility of a P. aeruginosa *ridA* mutant

Strain	Genotype	Swim zone^*a*^ (mm)	Swim zone + THI (mm)
DMPA4	Wild-type	21 ± 0.5^*b*^	20 ± 1^*b*^
DMPA7	*ridA*	11 ± 0.5	13 ± 0.5
DMPA14	*iscS*_(A548C)_	14 ± 0.5^*b*^	14 ± 0.5^*b*^
DMPA5	*dapA*_(A321G, A322G)_	17 ± 0.5^*b*^	17 ± 1^*b*^
DMPA13	*PA1559*_(ΔTCA188)_	17 ± 0.5^*b*^	18 ± 0.5^*b*^

a Swim halo diameters were measured in minimal medium with glucose (11 mM) and low agar (0.3%). Thiamine (THI) was present at 100 nM when indicated. Data are presented as the mean and standard deviation of the swimming halo diameter of three biological replicates grown at 37°C for 20 h. Values for each biological replicate were the average of two technical replicates.

b Indicates a significant difference in motility relative to the *ridA* mutant (*P* < 0.05).

10.1128/mbio.01071-22.3 TABLE S1 Spontaneous mutations restore motility of a P. aeruginosa *ridA* mutant. The diameter of swimming halos was measured in minimal medium with glucose (11 mM) and 0.3% agar. l-isoleucine (1 mM) was added as indicated. Data are presented as the mean diameter and standard deviation of the swim halo of three biological replicates grown at 37°C for 20 h. Values for each biological replicate were the average of two technical replicates. An asterisk (*) indicates a significant difference in motility relative to the *ridA* mutant (*P* < 0.05). Download Table S1, DOCX file, 0.01 MB. Copyright © 2022 Fulton et al. 2022 Fulton et al. https://creativecommons.org/licenses/by/4.0/ This content is distributed under the terms of the Creative Commons Attribution 4.0 International license.

### DapA^N108G^ variant restored growth and motility of the *ridA* mutant.

Suppressor mutant DMPA5 had an AA to GG substitution at bases 321 and 322 in the coding sequence of *dapA*/PA1010 (dihydropicolinate synthase; EC 4.3.3.7) resulting in a DapA^N108G^ variant. DapA from P. aeruginosa shares 56% protein sequence identity with DapA from S. enterica. In S. enterica, mutations in *dapA* also eliminated the growth defects of a *ridA* mutant (20). In S. enterica, DapA variants that suppressed *ridA* mutant phenotypes had, on average, a 50-fold lower specific activity than the wild-type enzyme. These variants increased metabolic flux toward threonine, ultimately decreasing the IlvA-mediated generation of 2AA (20, 21). Residue N108 is adjacent to Y107 (E. coli numbering), a conserved active site residue that significantly lowers DapA activity when disrupted (20, 22). Based on the S. enterica paradigm and the location of the suppressing substitution, we predicted the mechanism of suppression in the P. aeruginosa was similar to S. enterica and this allele was not pursued further.

### A mutation in *iscS* restored the growth of a P. aeruginosa *ridA* mutant.

Strain DMPA14 had a mutation in *iscS* (*PA3814*) that encoded a variant enzyme in which Q183, a conserved residue in the PLP-binding domain of IscS (Nfs1 in eukaryotes) homologs, was replaced with proline. The IscS^Q183P^ variant suppressed the growth defect of a *ridA* mutant in the presence of exogenous serine (Fig. 2C). Pseudomonas aeruginosa has three putative cysteine desulfurases (PA2062, PA3667, and PA3814). Of the three, PA3814 has the highest protein sequence identity (75%) to S. enterica IscS (STM2543) and lies in the *isc* operon. Therefore, this protein was assumed to be involved in the Fe-S cluster biosynthesis and was designated *iscS* in the genome annotation. The crystal structure of E. coli IscS, in addition to structural studies of S. enterica IscS, suggested a Q183P substitution could alter interactions in the PLP-binding domain and potentially affect enzymatic activity (23, 24).

### An IscS^Q183P^ variant partially suppressed a *ridA* mutant of S. enterica and was dominant.

**FIG 3:**
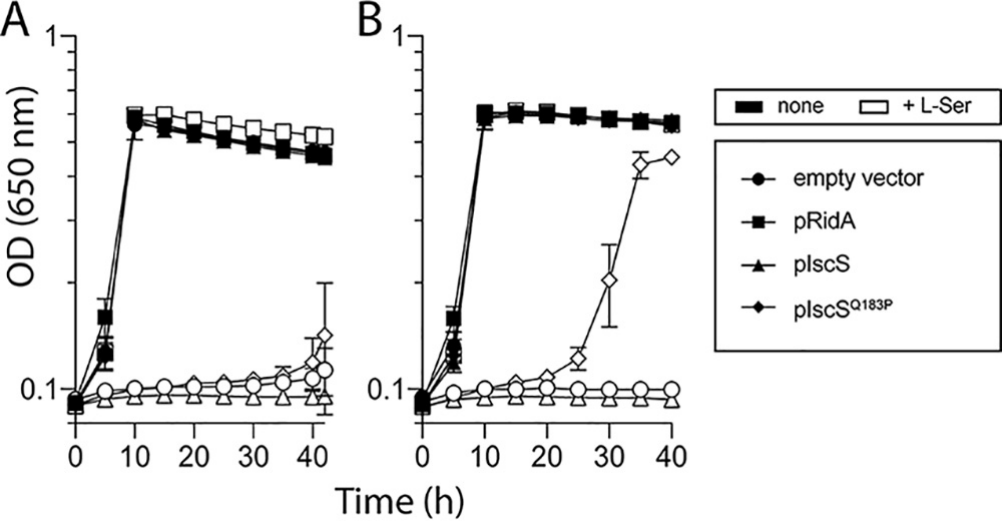
The *iscS*_(A548C)_ allele of *P. aeruginosa* in *trans* restores growth to an S. enterica *ridA* mutant with serine. Bacterial growth was measured as the change in optical density (650 nm) over time for an S. enterica *ridA* mutant with the pCV1 empty vector (circles), pDM1439 encoding _SE_RidA (squares), pDM1636 encoding _PA_IscS (triangles), or pDM1637 encoding _PA_IscS^Q183P^ (diamonds). Growth of the relevant strains was monitored in minimal glucose medium without l-arabinose (A) or with 0.2% l-arabinose (B) and no further additions (filled symbols), or 5 mM l-serine (open symbols). Error bars represent the standard deviation between three independent biological replicates.

The dominance of the *iscS*_(A548C)_ allele was tested in an S. enterica system. S. enterica strain DM12920 (*ridA*) was transformed with plasmids expressing _PA_IscS, _PA_IscS^Q183P^, _SE_RidA, or a vector-only control. The growth of the resulting strains was assessed in a minimal medium containing serine (Fig. 3). Several points were noted. First, when expressed in *trans*, _PA_IscS^Q183P^ allowed growth of the *ridA* mutant of S. enterica while _PA_IscS failed to restore growth. Second, the growth stimulation by _PA_IscS^Q183P^ required that expression of the gene was induced and had a lag of ~20 h. In contrast, _SE_RidA expressed in *trans* restored full growth without induction or a lag period. Finally, exogenous isoleucine restored growth to all strains as expected (unpublished data). These data showed that the _PA_IscS^Q183P^ variant was dominant with respect to its ability to suppress *ridA* growth defects. Further, the partial suppression of S. enterica *ridA* mutant phenotype by this variant revealed a common feature of 2AA stress in the two organisms.

### An IscS^Q183P^ variant had cysteine desulfurase activity *in vivo*.

**FIG 4:**
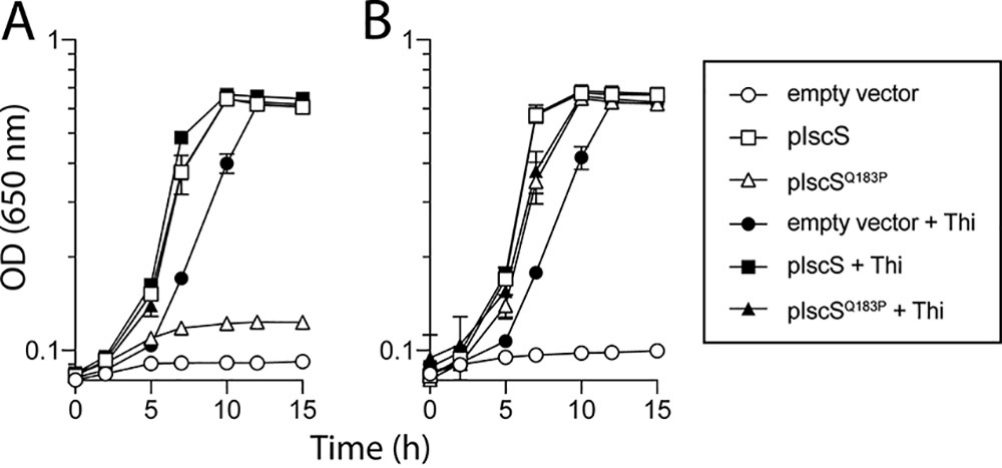
IscS^Q183P^ has cysteine desulfurase activity *in vivo*. S. enterica strain (DM5419), which is auxotrophic for thiamine and nicotinic acid (*iscR1*::MudJ) (25), was transformed with a pCV1 empty vector (circles), pDM1636 (squares), or pDM1637 (triangles), encoding _PA_IscS and _PA_IscS^Q183P^, respectively. The resulting strains were grown in minimal glucose medium with nicotinic acid alone (open shapes) or with nicotinic acid and 100 nM thiamine (filled shapes). l-arabinose (0.2%) was absent (A) or present (B) in the medium to induce expression of the plasmid-borne gene. Error bars represent the standard deviation between three independent biological replicates.

The results above raised the question of whether the _PA_IscS^Q183P^ variant retained cysteine desulfurase activity (EC 2.8.1.7). P. aeruginosa has three IscS homologs and the consequences of lacking PA3814 and potential overlap with the other paralogs were not known. In contrast, phenotypes of S. enterica strains lacking *iscS* have been described and, thus, were exploited to determine if _PA_IscS^Q183P^ had cysteine desulfurase activity *in vivo.* IscS was required for thiamine biosynthesis in S. enterica. Strain DM5419 (*iscR1*::MudJ) required thiamine and nicotinic acid due to polar effects on the downstream genes *iscS* and *iscA*, respectively (25). Importantly, a functional IscS in *trans* eliminates the thiamine requirement of this strain. DM5419 was transformed with plasmids encoding _PA_IscS, _PA_IscS^Q183P^, or an empty vector. Growth of the resulting strains, with and without induction of the plasmid-borne gene, was assessed in liquid minimal media supplemented with nicotinic acid (Fig. 4). _PA_IscS restored growth of DM5419 in the absence of thiamine whether or not expression was induced. _PA_IscS^Q183P^ also restored growth in the absence of thiamine, but only when expression of the relevant gene was induced. These results showed that IscS^Q183P^ retained cysteine desulfurase activity *in vivo*, and suggested it had lower activity than wild-type IscS.

The interpretation that _PA_IscS^Q183P^ retains cysteine desulfurase activity was supported by assaying the Fe-S cluster protein succinate dehydrogenase (SDH) in both S. enterica and P. aeruginosa. The activity of SDH was dependent on cysteine desulfurase activity to generate its Fe-S cluster and could, thus, be used as a proxy for IscS activity. In S. enterica strain DM5419 (*iscR1*::MudJ) expressing _PA_IscS or _PA_IscS^Q183P^ grown in a minimal medium, SDH activity (measured as the change in absorbance at 600 nm [ΔA_600_]/min/mg protein) was 4.2 ± 1.0 and 3.9 ± 0.5, respectively. SDH activity in the same strain with an empty vector was 1.6 ± 0.3, which reflects the activity of SDH in the absence of a functional IscS. SDH activity was also determined in crude extracts of the *ridA* mutant of P. aeruginosa and the suppressor strain with IscS^Q183P^ (Table 3). In a rich medium (where the loss of *ridA* has no detrimental effect), there was significantly less SDH activity when IscS^Q183P^ was present compared to wild-type IscS. These data supported the conclusion that IscS^Q183P^ had less desulfurase activity than the parental protein, as suggested by the complementation data in Fig. 4. Further, the SDH activity of both strains in a minimal medium was increased by supplementation with isoleucine. These data were consistent with a decrease in 2AA stress due to the allosteric inhibition of serine/threonine dehydratase by isoleucine (7).

**TABLE 3:** IscS^Q183P^ retained cysteine desulfurase activity

Condition	SDH activity^*a*^ (ΔA_600_/min/mg protein)	
Medium	*ridA*	*ridA iscS*_(A548C)_
LB	13.9 ± 2.0	10.9 ± 1.5^*b*^
Minimal	3.7 ± 0.6	5.6 ± 0.4
Minimal + Isoleucine	8.1 ± 1.0^*c*^	6.7 ± 1.0^*c*^

a Data shown are the means of two biological and four technical replicates using the SDH assay described in Materials and Methods. The indicated strains were grown in the stated medium with succinate as the sole carbon source in minimal medium and isoleucine added to 1 mM.

b Indicates a significant difference in activity (*P* > 0.05) compared to the *ridA* mutant grown in the same medium.

c Indicates a significant difference in activity (*P* > 0.05) compared to the same strain grown in the absence of isoleucine.

### IscS^Q183P^ had a unique mechanism of suppression.

**FIG 5:**
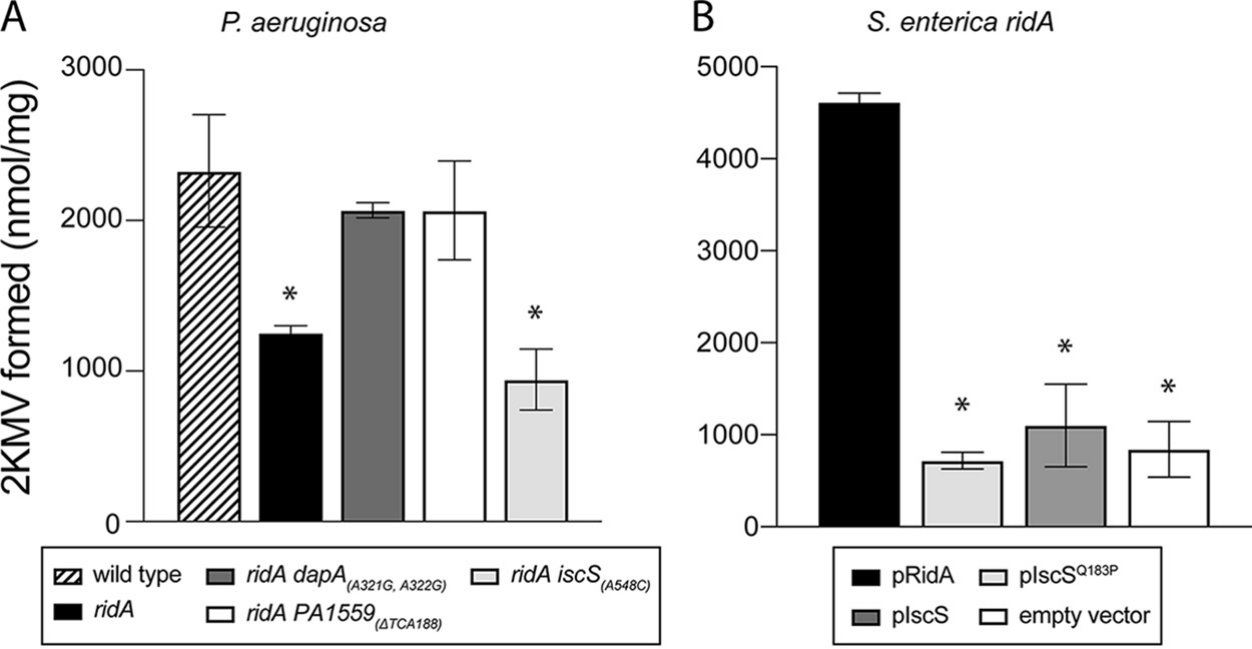
IscS^Q183P^ did not reduce 2AA accumulation. The relevant strains of P. aeruginosa and S. enterica were grown to full density in minimal glucose medium and transaminase B (IlvE) activity was determined. Strains used were (A) wild-type P. aeruginosa strain (stripes), a *ridA* mutant (black), and three spontaneous *ridA* suppressor mutants, *PA1559*_(ΔTCA188)_ (white), *dapA*_(A321G, A322G)_ (dark gray), and *iscS*_(A548C)_ (light gray) and (B) *ridA* mutant of S. enterica was transformed with plasmids encoding _SE_RidA (black), _PA_IscS (dark gray), _PA_IscS^Q183P^ (white) or empty vector control (light gray). Error bars represent the standard deviation of three independent biological replicates. An asterisk (*) indicates a significant difference in IlvE activity compared to wild-type P. aeruginosa (A) or S. enterica *ridA* with a plasmid encoding _SE_RidA (B) (*P* < 0.05).

Several genetic and nutritional conditions that suppress phenotypes of an S. enterica *ridA* mutant have been characterized. In all cases, the mechanism of suppression was to decrease the generation of 2AA or nutritionally bypass a key enzyme damaged by 2AA (18, 20, 26–28). Transaminase B (IlvE) is a target of 2AA damage, and its activity has been used as a proxy for 2AA levels in multiple organisms, including P. aeruginosa (7). IlvE activity was assayed in three suppressor mutants of P. aeruginosa (Fig. 5A). As expected, the *ridA* mutant had significantly lower transaminase B activity than the parental strain. The suppressing alleles of *dapA* and *PA1559* restored transaminase B activity to levels found in the wild-type strain. Interestingly, the *iscS*_(A548C)_ allele did not increase transaminase B activity. These data supported the hypothesis that this mutation suppressed the *ridA* phenotypes by a mechanism not previously described. Additional support for this conclusion was provided by the heterologous S. enterica system (Fig. 5B). In this case, transaminase B activity was assayed in a *ridA* mutant carrying an empty vector or expressing _SE_RidA, _PA_IscS, or _PA_IscS^Q183P^. Neither _PA_IscS nor _PA_IscS^Q183P^ restored transaminase B activity in the S. enterica strains (Fig. 4). In total, these data supported the unique scenario in which the IscS^Q183P^ variant allowed a *ridA* mutant to grow in the presence of 2AA stress.

### Thiamine supplementation restored growth to *ridA* mutants.

The ability of a variant of IscS to suppress *ridA* defects in both P. aeruginosa and S. enterica *ridA* mutants without lowering 2AA levels suggested an exciting possibility. We hypothesized that IscS was (i) a target of damage by 2AA and (ii) the 2AA-mediated decrease in IscS activity was primarily responsible for the growth defect of a P. aeruginosa *ridA* mutant. If this were the case, growth of the *ridA* mutant might be restored by exogenous thiamine because it should bypass a presumed nutritional consequence of decreased cysteine desulfurase activity of IscS.

**FIG 6:**
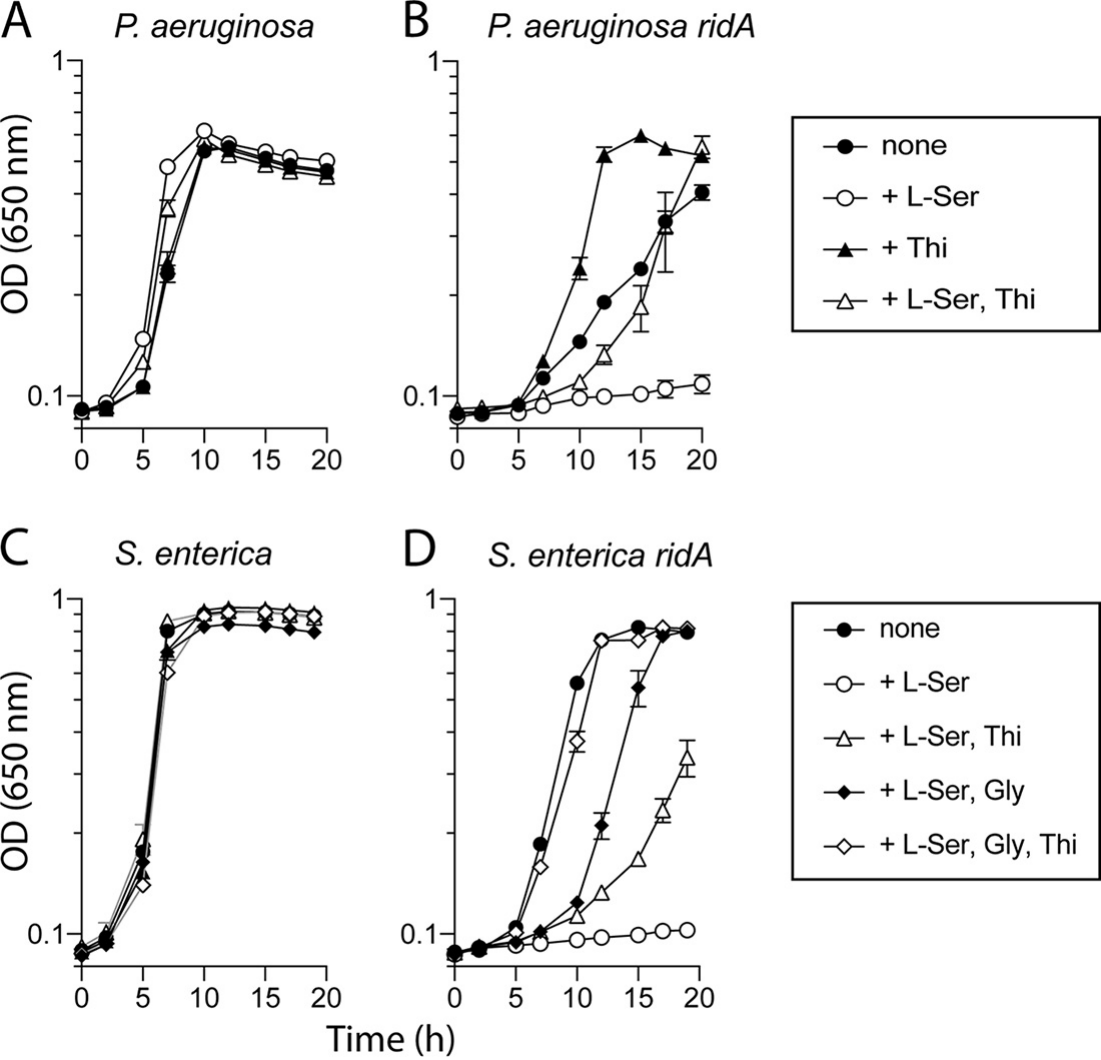
Exogenous thiamine restores growth of *ridA* mutants in P. aeruginosa and S. enterica. Growth was measured for P. aeruginosa wild-type (A) and P. aeruginosa *ridA* (B), along with S. enterica wild-type (C) and S. enterica *ridA* (D). P. aeruginosa strains were grown in minimal glucose medium with no additions (closed circles), minimal glucose supplemented with 0.5 mM l-serine (open circles), 100 nM thiamine (closed triangles), or both l-serine and thiamine (open triangles). S. enterica strains were grown in minimal glucose medium with no additions (closed circles), 5 mM l-serine (open circles), l-serine and 100 nM thiamine (open triangles), l-serine and 1 mM glycine (closed diamonds), or l-serine, thiamine, and glycine (open diamonds). Error bars represent the standard deviation between three independent biological replicates.

The growth of wild-type and *ridA* mutant strains was monitored in minimal glucose medium supplemented with serine and/or thiamine (Fig. 6A and B). Consistent with the above scenario, thiamine significantly increased growth of the *ridA* mutant in a minimal medium with or without serine present. Growth was not completely restored by thiamine in the presence of serine. This result suggested either there was an additional target(s) of 2AA that contributed to the growth phenotype, or that the consequence of decreased IscS activity extended beyond thiamine synthesis.

In S. enterica, serine hydroxymethyltransferase (GlyA, E. C. 2.1.2.1) is the critical target of 2AA and glycine almost entirely alleviates the growth defect of a *ridA* mutant (18). The data in Fig. 3 suggested thiamine biosynthesis might also be compromised in an S. enterica *ridA* mutant due to 2AA-mediated damage of IscS. Growth experiments with S. enterica in minimal glucose medium supplemented with various combinations of serine, glycine, and thiamine supported this conclusion (Fig. 6C). As expected, serine prevented growth and the addition of glycine restored it considerably (18). The addition of thiamine improved growth in the presence of serine, but not to the level allowed by glycine, while the addition of both thiamine and glycine fully restored growth of the *ridA* mutant in the presence of serine to that in minimal medium alone. These data identified the second target of 2AA damage in S. enterica that contributes to the growth defect of a *ridA* mutant.

### The IscS^Q183P^ variant had decreased susceptibility to 2AA damage *in vivo*.

**FIG 7:**
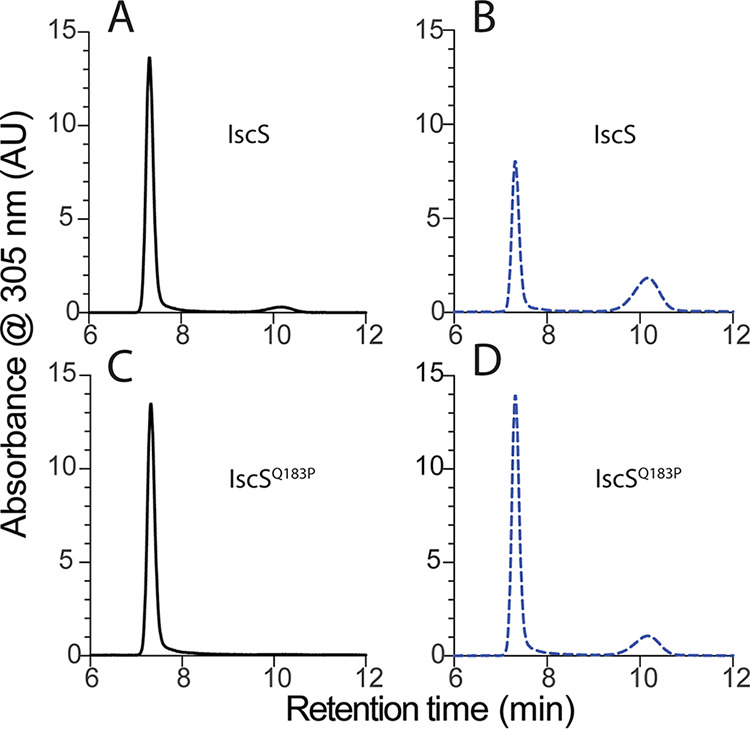
IscS was sensitive to attack by 2AA. IscS and IscS^Q183P^ were each purified from two S. enterica host strains, wild-type and a *ridA* mutant. Cofactors were released from each sample, separated by HPLC, and visualized by monitoring absorbance at 305 nm in arbitrary units (AU). Shown are the profiles of cofactors released from IscS purified from wild-type (A) and a *ridA* mutant (B) and those from IscS^Q183P^ purified from wild-type (C) and a *ridA* mutant (D). The peak with a retention time of ~7 min was PLP and that with a retention time of ~10 min was pyruvate/PLP. Peak assignment was based on retention time, UV-vis spectra, and coinjection with authentic species (Fig. S2). Absorbance was monitored at 305 nm to capture both species. Data shown are an average of two technical replicates.

Genetic experiments above showed IscS was a critical target of 2AA damage in P. aeruginosa. We considered a scenario in which the IscS^Q183P^ variant restored growth because it was less susceptible to a 2AA attack than wild-type IscS. To assess this possibility, we used an S. enterica system that can monitor 2AA-dependent damage that occurs *in vivo* (15, 17, 29). _PA_IscS and _PA_IscS^Q183P^ proteins were overexpressed and purified from two different S. enterica strains, *ridA* and wild-type, resulting in four protein samples. The four relevant strains were grown in minimal medium with arabinose and IPTG to ensure protein overexpression, serine to induce 2AA stress, and glycine to allow growth in the presence of such stress. When 2AA attacks an active site PLP, a pyruvate/PLP adduct can be extracted from the damaged enzyme by treatment with base (15, 27). The four protein samples were treated with base, and the cofactors released from each were separated by high-performance liquid chromatography (HPLC). Several points were noted from the data (Fig. 7 and Table 4). Importantly, IscS purified from the *ridA* mutant released significant pyruvate/PLP, indicating that IscS was a target of 2AA attack *in vivo* (Fig. 7B). In contrast, IscS purified from a wild-type strain released predominately PLP, with a barely detectable pyruvate/PLP peak (Fig. 7A). The presence of a small but detectable level of pyruvate/PLP was consistent with results for some other protein targets of 2AA, which showed a low level of 2AA stress even in the presence of RidA (29).

**TABLE 4:** IscS was a target of 2AA

		Peak area^*a*^		% released cofactor^*b*^	
Host strain	Protein^*c*^	PLP	Pyruvate/PLP	PLP	Pyruvate/PLP
*ridA* mutant	IscS	93.5 ± 1.4	64.9 ± 10.7	59	41
*ridA* mutant	IscS^Q183P^	168.1 ± 35	37.1 ± 3.2	82	18
wild-type	IscS	165.1 ± 4.1	9.4 ± 2.8	5	95
wild-type	IscS^Q183P^	162.6 ± 5.3	ND	100	0

a Peak area is in arbitrary units (AU).

b Percentage of the released cofactor is reported as the ratio of the indicated cofactor as the numerator over the total cofactor as the denominator.

c IscS and IscS^Q183P^ proteins were each purified from two S. enterica host strains, wild-type, and a *ridA* mutant. Cofactors were released by treatment with base and separated by HPLC while monitoring absorbance at 305 nm in arbitrary units. Absorbance was monitored at 305 nm to capture both species. Therefore, peak area could be used to represent relative ratios but not the absolute concentration of each cofactor. Data were extracted from the chromatograph and peaks called and areas were determined by the HPLC instrument. Data shown are the average and standard deviation of two technical replicates. “ND” indicates that a peak was not detected at 305 nm.

10.1128/mbio.01071-22.2 FIG S2 Standards confirm peaks as PLP and pyruvate/PLP. Authentic pyruvate/PLP was generated and purified as described in Materials and Methods. Due to the instability of the pyruvate/PLP, over time this sample contained both PLP and pyruvate/PLP and thus provided a standard for both cofactors. Cofactors released from IscS purified from a *ridA* mutant (A) and wild-type (B), along with IscS^Q183P^ purified from a *ridA* mutant (C) and wild-type (D) were separated by HPLC. In each case, the sample was run alone (solid lines) or with a coinjection of a sample containing authentic pyruvate/PLP and PLP (blue dashed lines). The peak at ~7 minutes corresponds to PLP, and the peak at ~10 minutes corresponds to pyruvate/PLP. The structure of the relevant molecules, PLP and pyruvate/PLP is shown. Download FIG S2, JPG file, 0.6 MB. Copyright © 2022 Fulton et al. 2022 Fulton et al. https://creativecommons.org/licenses/by/4.0/ This content is distributed under the terms of the Creative Commons Attribution 4.0 International license.

Analysis of the IscS^Q183P^ variant supported the hypothesis that this protein was less susceptible to 2AA damage than the wild-type protein. IscS^Q183P^ purified from a *ridA* mutant released significantly less pyruvate/PLP than the wild-type protein (Fig. 7D). The calculated areas (in arbitrary units) of the pyruvate/PLP peaks from the IscS^Q183P^ and IscS samples were 37 and 65, respectively (Table 4). Further, the IscS^Q183P^ protein sample purified from the wild-type strain did not release detectable pyruvate/PLP. Taken together, these data supported the conclusions that IscS was a target of 2AA, and further, the IscS^Q183P^ variant was less sensitive to attack by 2AA than the wild-type protein.

In contrast to pyruvate/PLP, similar amounts of PLP were released from three of the four IscS protein samples. However, when wild-type IscS protein was purified from *ridA* (i.e., in the presence of 2AA), the amount of released PLP decreased significantly. The decreased concentration of PLP was reflected by a decrease in peak area from ~165 to ~93 arbitrary units. The significance of this decrease was unclear, but it suggested the effect of 2AA was more far-reaching than indicated simply by measuring the pyruvate/PLP adduct. This result further supported the notion that the IscS^Q183P^ variant was less affected by 2AA stress than IscS. The ratios of cofactors released from each sample purified from the *ridA* mutant supported this conclusion. In the IscS sample, pyruvate/PLP made up 40% of the released cofactor, while in the IscS^Q183P^ sample this number was 18%. Thus, both concentration and percentage measurements of the pyruvate/PLP adduct supported the conclusion that the IscS^Q183P^ variant was less sensitive to 2AA than the wild-type protein. The precise correlation between damage, enzymatic activity, and PLP occupancy of these enzymes with their physiological significance will require additional work.

## DISCUSSION

Mutants of P. aeruginosa lacking RidA have observable phenotypes that reflect the consequences of 2AA accumulation (7). Three mutations that suppress these phenotypes were isolated and discussed herein. In the context of the RidA paradigm, phenotypic suppression can be generated by conditions, or mutations that (i) decrease or prevent the generation of 2AA, (ii) increase quenching of 2AA, (iii) bypass the damaged target that is responsible for preventing growth under a relevant condition, or (iv) prevent damage to a key target enzyme. Conditions that allow the former three mechanisms have been described (18, 20, 26–28). Here, we reported a mutation that suppresses the *ridA* phenotype in P. aeruginosa by generating a target protein variant with decreased sensitivity to 2AA.

Three spontaneous mutations were isolated that suppressed *ridA* mutant phenotypes in P. aeruginosa: (i) an in-frame deletion in *PA1559*, (ii) a dinucleotide polymorphism in *dapA*, and, (iii) a single nucleotide polymorphism in *iscS.* PA1559, or CprA (catatonic peptide resistance), is a hypothetical protein suggested to have a role in PhoPQ-mediated polymyxin resistance (30, 31). The role of this locus in 2AA stress was not pursued in this study. The lesion in *dapA* was predicted to act by the mechanism characterized for lesions in the same locus in S. enterica. Briefly, lowering the activity of DapA increases flux to threonine/isoleucine which reduces 2AA formation by IlvA (20, 21). Suppressing lesions in *dapA* and *PA1559* restored transaminase B (IlvE) activity in the P. aeruginosa *ridA* mutant, indicating they had reduced endogenous levels of 2AA.

In contrast, the suppressing mutation in *iscS* (encoding IscS^Q183P^) restored growth to a *ridA* mutant without lowering 2AA levels. The *isc* (iron-sulfur cluster) genes are involved in iron-sulfur cluster assembly and trafficking sulfur to various enzymes and tRNAs. IscS (EC 2.8.1.7) is a fold-type I PLP-dependent enzyme that mobilizes sulfur by desulfurization of cysteine to yield an IscS-bound persulfide and alanine (32). Interestingly, cysteine desulfurases generate 2AA as an obligate intermediate, but in the catalytic mechanism of IscS/SufS, the enamine species is converted to l-alanine in the active site of the enzyme (33). Thus, this enzyme would not contribute to 2AA stress in a cell. Mutations in *iscS* that affect the biosynthesis of thiamine, biotin, NAD, isoleucine/valine, and molybdopterin, in addition to iron homeostasis and tRNA activation have been reported (25, 34–37).

The results of this work support the hypothesis that IscS is a target of 2AA damage and that damage to this enzyme is primarily responsible for the growth defects of a P. aeruginosa *ridA* mutant. Consistent with this scenario, thiamine restored the growth of a P. aeruginosa *ridA* mutant in the presence of serine. Additionally, the suppressing variant, IscS^Q183P^, is less sensitive to 2AA attack than the wild-type IscS *in vivo* and retains at least some of its native cysteine desulfurase activity. Although it is not clear how attack by 2AA is impaired, it was previously shown that residues at position 183 play a role in stabilizing the unprotonated phenolic oxygen (O3’) of PLP by serving as a hydrogen bond donor (38). Consistently, the presence of a glutamine residue at position 183 is widely conserved in IscS and its homologs (39). Thus, the data here, together with those in the literature, favor a situation in which a substitution at residue 183 could affect 2AA susceptibility by altering the structure of the PLP-binding domain of IscS or electrophilicity of the PLP cofactor. Further biochemical and structural studies are needed to clarify the mechanism of resistance.

In total, the results herein from both *in vivo* and *in vitro* experiments support the conclusion that the growth defect of a P. aeruginosa *ridA* mutant is primarily caused by 2AA-dependent damage to the cysteine desulfurase IscS. With this work, P. aeruginosa becomes the second bacterium (in addition to S. enterica) in which the primary mechanism of growth inhibition by 2AA stress has been determined. This work also furthered our understanding of 2AA stress in S. enterica by identifying IscS, in addition to GlyA, as a target of damage with nutritional consequences. These results provide further evidence that 2AA targets are conserved across species, but phenotypic outcomes associated with 2AA stress are organism-specific and are due to differences in the metabolic architecture of each organism. These differences ultimately dictate the enzymatic activity or metabolic pathway(s) that most affect the stability of the network. It is these components that can lead to detectable growth consequences when disrupted. The ability of a single substitution in IscS to render the enzyme significantly less sensitive to 2AA damage was unexpected and will inform further efforts to understand the mechanism by which endogenous 2AA attacks cellular enzymes. This result raised questions about not only determinants of sensitivity, but how selective pressure may act to decrease sensitivity, potentially at the expense of enzymatic activity.

## MATERIALS AND METHODS

### Bacterial strains, media, and chemicals.

Bacterial strains used in this study are listed in Table 1. Pseudomonas aeruginosa PAO1 wild-type (MPAO1) and *ridA* (PW9994 *ridA*-F05::ISphoA/hah) were obtained from the transposon mutant library collection (40). Salmonella enterica serovar Typhimurium LT2 strains were available, or derivatives of those in the laboratory strain collection.

P. aeruginosa strains were grown at 37°C and Lysogeny broth (LB) was used as a rich medium. M9 salts (20 mM NH_4_Cl, 12 mM Na_2_HPO_4_, 22 mM KH_2_PO_4_, 1.0 mM NaCl, 1 mM MgSO_4_) with trace minerals (41) and glucose (11 mM) or succinate (20 mM) was used as a minimal medium (42, 43). Supplements were added as indicated; isoleucine (1 mM) and serine (0.05 mM). Chemicals were purchased from MilliporeSigma (Sigma-Aldrich, St. Louis, MO).

For the growth of S. enterica, Difco nutrient broth (NB) (8 g/L) containing NaCl (5 g/L) was used as a rich medium. The minimal medium consisted of no-carbon E salts (NCE) with 1 mM magnesium sulfate (42), trace elements (41), and glucose (11 mM) as the sole carbon source. Difco BiTek agar (15 g/L) was added to make a solid growth medium. Serine (5 mM) and isoleucine (1 mM) were added to a minimal medium as indicated. Ampicillin was added to the rich and minimal medium at a concentration of 150 mg/L and 15 mg/L, respectively. Amino acids and antibiotics were purchased from Sigma-Aldrich (St. Louis, MO).

### P. aeruginosa mutant isolation.

Independent cultures (1 mL) of DMPA7 were grown overnight in LB at 37°C with shaking. Fully grown cultures were centrifuged, and the pellet was resuspended in saline (1 mL). An aliquot of the cell suspension (100 μL) was spread on a minimal medium with serine (0.5 mM) and incubated at 37°C for ~72 h, at which time colonies were visible. The putative suppressor mutants were streaked for isolation on a solid minimal M9 medium with serine and were phenotypically characterized.

In a second selective condition, aliquots of a cell suspension (10 μL) were inoculated into motility agar by gently piercing the top of the agar and expelling cells. After 5 days of incubation at 37°C, asymmetrical outgrowths from the center motility halo were noted. A sterile toothpick recovered cells furthest from the center inoculation point. This toothpick was used to (i) inoculate a second motility plate and (ii) streak for isolated colonies on a solid M9 minimal medium. Mutants that showed higher motility than the parental *ridA* strain (DMPA7) were characterized further.

### Phenotypic growth analysis.

Growth in the solid medium was evaluated by patching strains to rich medium (LB for P. aeruginosa and NB for S. enterica), incubating plates overnight at room temperature, and replica printing to agar plates with the relevant nutrients. Alternatively, 1 mL cultures were grown in rich medium overnight shaking at 37°C, pelleted, and resuspended in an equal volume of saline before embedding the cell suspension (100 μL) in 4 mL of soft agar overlaid on a solid medium. Nutrients were spotted on soft agar, plates were dried for 15 min at room temperature, and incubated overnight at 37°C.

Growth in liquid medium was assessed using a BioTek Elx808 microtiter plate reader following optical density at 650 nm (OD_650_) at 37°C with a slow shaking speed as described (7). Overnight cultures of P. aeruginosa or S. enterica in biological triplicate were grown in LB medium at 37°C, pelleted, and resuspended in an equal volume of saline. Aliquots (5 μL) of the cell suspension inoculated the relevant medium (195 μL) and growth was monitored for 24 h. Growth curves were plotted using GraphPad Prism (version 7.0).

### Motility.

M9 motility medium with 0.3% Bacto-agar (Difco) was prepared as described (7, 43). Molten medium (25 mL) was poured into petri dishes and allowed to sit for 6 h at room temperature before use. Triplicate cultures were grown overnight in LB at 37°C. Cultures were centrifuged, and the pellet was resuspended in a volume of saline to generate an OD_650_ of 0.3. Each bacterial suspension (1 μL) was inoculated into a motility plate by gently piercing the top of the agar and expelling the cells. Plates were dried for 15 min, then incubated at 37°C for 20 h or longer in a sealed container to maintain constant moisture. The diameter of each swimming halo was measured and reported in millimeters (mm) and/or as a percentage of that produced by the wild-type strain.

### Transaminase B assay.

Overnight cell culture from rich medium (250 μL) was inoculated into 25 mL of minimal medium with indicated additions and incubated at 37°C with shaking overnight. The cells were harvested by centrifugation and washed once with NCE (10 mL). Cell pellets were frozen at −80°C until use. Cell pellets were resuspended in 1.0 mL of 50 mM potassium phosphate, pH 7.5, and lysed using a Constant Systems Limited One Shot (United Kingdom) system by passing cells through the disrupter one time with the pressure set to 145 MPa. Unbroken cells and debris were pelleted at 17.0 × *g* for 20 min at 4°C. The protein concentration of the resulting cell extract was estimated using a bicinchoninic assay reagent kit (Pierce, Rockford, Ill.).

The protocol for the transaminase B assay has been described (7, 44). Briefly, a 50 μL aliquot of cell extract was added to a reaction mixture that contained PLP (50 μM) and α-ketoglutarate (10 mM) in potassium phosphate buffer (50 mM, pH 7.5) to a total volume of 200 μL. After equilibrating at 37°C for 10 min, the reaction was initiated by the addition of l-isoleucine (20 mM final concentration) and allowed to proceed for 20 min at 37°C. The reaction was stopped with 0.3% 2,4-dinitrophenyl-hydrazine (DNPH, 200 μL) to derivatize product 2-ketomethylvalerate (2KMV), forming a chromophore with absorbance at 540 nm. Results of the assay are reported in nmol 2-keto-3-methylvalerate (2KMV) formed/mg protein, based on a standard curve generated from known quantities of 2KMV similarly derivatized with DNPH. Data are presented as the mean of three biological replicates and error bars represent the standard error of the mean. Statistical significance (*P* < 0.05) was determined by conducting a one-way analysis of variance (ANOVA) and Tukey’s posttest using GraphPad Prism (version 7.0c).

### Next-generation sequencing and data analysis.

Genomic DNA was extracted from the relevant strains using the Monarch genomic DNA purification kit (New England BioLabs). Libraries were constructed using Nextera™ DNA Flex library kit and analyzed using the iSeq 100 System (Illumina). Genomic sequence reads were realigned and mapped to the published PAO1 genome using Geneious software (version 10.1.2). High-frequency single-nucleotide polymorphisms (SNPs) were detected and the respective impact on each coding sequence was predicted. SNPs of interest were confirmed by Sanger sequencing of PCR amplification of the relevant gene.

### Purification of proteins from S. enterica strains.

IscS-His_6_ and IscS^Q183P^-His_6_, encoded on pDM1684 and pDM1685, respectively, were purified from two S. enterica strains containing arabinose inducible T7 polymerase (45). The isogenic strains had (DM13509) or were lacking (DM17050) a functional RidA. Overnight cell cultures (10 mL) grown on SB supplemented with ampicillin (150 μg/mL) were inoculated into each of two Fernbach flasks (2.8 liters) containing 1.5 liters of minimal glycerol medium with ampicillin (15 μg/mL) supplemented with glycine (1 mM) and pyridoxine (50 μM). The resulting cultures were grown at 37°C with shaking to an OD_650_ of 0.5 before induction with arabinose (0.1%) and IPTG (100 μM), and the addition of l-serine (5 mM) and additional glycine (1 mM). Cultures were then grown at 23°C with shaking for 18 h. Cells were harvested by centrifugation at 5000 × *g* for 15 min. Cell pellets were resuspended in 2 mL/g cell weight of binding buffer (potassium phosphate pH 7.4 [50 mM], NaCl [150 mM], and imidazole [20 mM]). Lysozyme (2 mg/mL) and DNase (125 μg/mL) were added, and the cell suspension was placed on ice for 20 min. Cells were mechanically lysed using a Constant Systems Limited One Shot (United Kingdom) at 145 MPa. PMSF (1 mM) was added to the lysate, which was then clarified by centrifugation at 48000 × *g* for 45 min and filtered through a PVDF filter (0.45 μm pore size). The filtered lysate was loaded onto 5 mL HisTrap HP Ni-Sepharose columns and washed with binding buffer (5 column volumes). Bound protein was eluted by increasing the concentration of imidazole from 20 mM to 500 mM over 10 column volumes. Purified protein was concentrated using a centrifugal filter with a molecular weight cutoff of 30,000 kDa (Millipore). The concentrated protein was then moved into potassium phosphate buffer (50 mM, pH 7.4) containing NaCl (150 mM) and glycerol (10% wt/vol) by buffer exchange using a PD-10 desalting column (GE Healthcare). Densitometry showed that purified proteins were ~80% pure (Fig. S1).

10.1128/mbio.01071-22.1 FIG S1 IscS and IscS^Q183P^ purified from *ridA+* and *ridA-* S. enterica. IscS or IscS^Q183P^ samples purified from S. enterica SB300 *ridA+* (DM13509) or *ridA-* (DM17050). Samples were separated by electrophoresis on a 12% polyacrylamide gel, stained with Coomassie, and imaged using AnalytikJena UVP ChemStudio. Densitometry analysis was performed using VisionWorks software version 8.22.18309.10577. BioRad Precision Plus Kaleidoscope Protein Ladder was run in the left-most lane. Samples (from left to right) are IscS from wild-type, IscS from *ridA* mutant, IscS^Q183P^ from wild-type, and IscS^Q183P^ from *ridA* mutant. 4 μg of protein was loaded for each sample. Download FIG S1, TIF file, 1.8 MB. Copyright © 2022 Fulton et al. 2022 Fulton et al. https://creativecommons.org/licenses/by/4.0/ This content is distributed under the terms of the Creative Commons Attribution 4.0 International license.

### Succinate dehydrogenase assays.

P. aeruginosa strains were grown in 25 mL of minimal succinate medium with or without l-isoleucine (1 mM) supplemented with thiamine (200 nM) or LB to mid-log-phase (OD_650_ ~0.5). S. enterica strains were grown in 5 mL minimal glucose medium supplemented with nicotinic acid (20 μM) and thiamine (200 nM) or NB with ampicillin added to both media. Cells were pelleted, washed with an equal volume of 50 mM cold potassium phosphate buffer pH 7.4 (50 mM), and frozen at −80°C for no longer than 48 h until use. Frozen pellets were thawed on ice and resuspended in 1 mL of cold potassium phosphate buffer before being mechanically lysed using a Constant Systems Limited One Shot (United Kingdom) at 210 Mpa. The cell lysate was clarified by centrifugation at 12000 × *g* for 15 s. Succinate dehydrogenase (SDH) was assayed according to a previously described method (25). The linear range was determined for each strain under each condition and specific activities were calculated as ΔA_600_/min/mg protein. SDH activity measured in boiled extracts was subtracted from the activity of each sample.

### Characterization of cofactor content.

Cofactors were released from each preparation of IscS and IscS^Q183P^ as described previously (15, 17, 29). KOH (30 mM final concentration) was added to 1.5 nmol purified protein in a 100 μL reaction and incubated at room temperature for 10 min. Protein was then precipitated with 10% trifluoroacetic acid (30 μL), resulting in a final volume of 130 μL. The precipitate was removed by centrifugation (17,000 × *g* for 3 min) and the supernatant was filtered through a 0.45 μm centrifugal tube filter (Costar 8170). Cofactors were separated by HPLC using a Shimadzu HPLC equipped with a Luna C18 column (Phenomenex) using a two-step isocratic method with a flow rate of 0.8 mL/min: 0 to 5 min with 100% buffer A (0.06% vol/vol trifluoroacetic acid) and 5 to 18 min with methanol and buffer A (3:97). The column was washed with methanol and buffer A (60:40) for 10 min between each run. Eluant was monitored at 305 nm using a photodiode array detector (Shimadzu SPD-M20A). Authentic PLP and pyruvate/PLP were used as standards to allow peak assignment. Pyruvate/PLP was synthesized as described previously (46), purified by HPLC, and concentrated.
