# Supplementary material for: The Cysteine Desulfurase IscS Is a Significant Target of 2-Aminoacrylate Damage in Pseudomonas aeruginosa
Source: mBio. 2022 Jun 2;13(3):e01071-22. doi: 10.1128/mbio.01071-22 (PMC9239102; doi:10.1128/mbio.01071-22)
Supplement: TABLE S1 [file mbio.01071-22-s0001.docx]

**Table S1**: Spontaneous mutations restore motility of a *P. aeruginosa ridA* mutant.

| Strain | Relevant Genotype | Swim diameter (mm) | Swim diameter + Ile (mm) |
| --- | --- | --- | --- |
| DMPA4 | Wild type | 18 ± 0.5* | 19 ± 1 |
| DMPA7 | *ridA* | 8 ± 0.5 | 19 ± 1 |
| DMPA14 | *ridA iscS_(A548C)_* | 12 ± 0.5* | 20 ± 0.5 |
