# Supplementary figures and images for: The Cysteine Desulfurase IscS Is a Significant Target of 2-Aminoacrylate Damage in Pseudomonas aeruginosa
Source: mBio. 2022 Jun 2;13(3):e01071-22. doi: 10.1128/mbio.01071-22 (PMC9239102; doi:10.1128/mbio.01071-22)

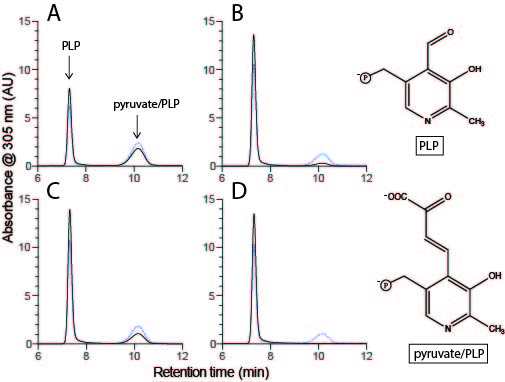

Supplement: FIG S2 [file mbio.01071-22-s0003.jpg]

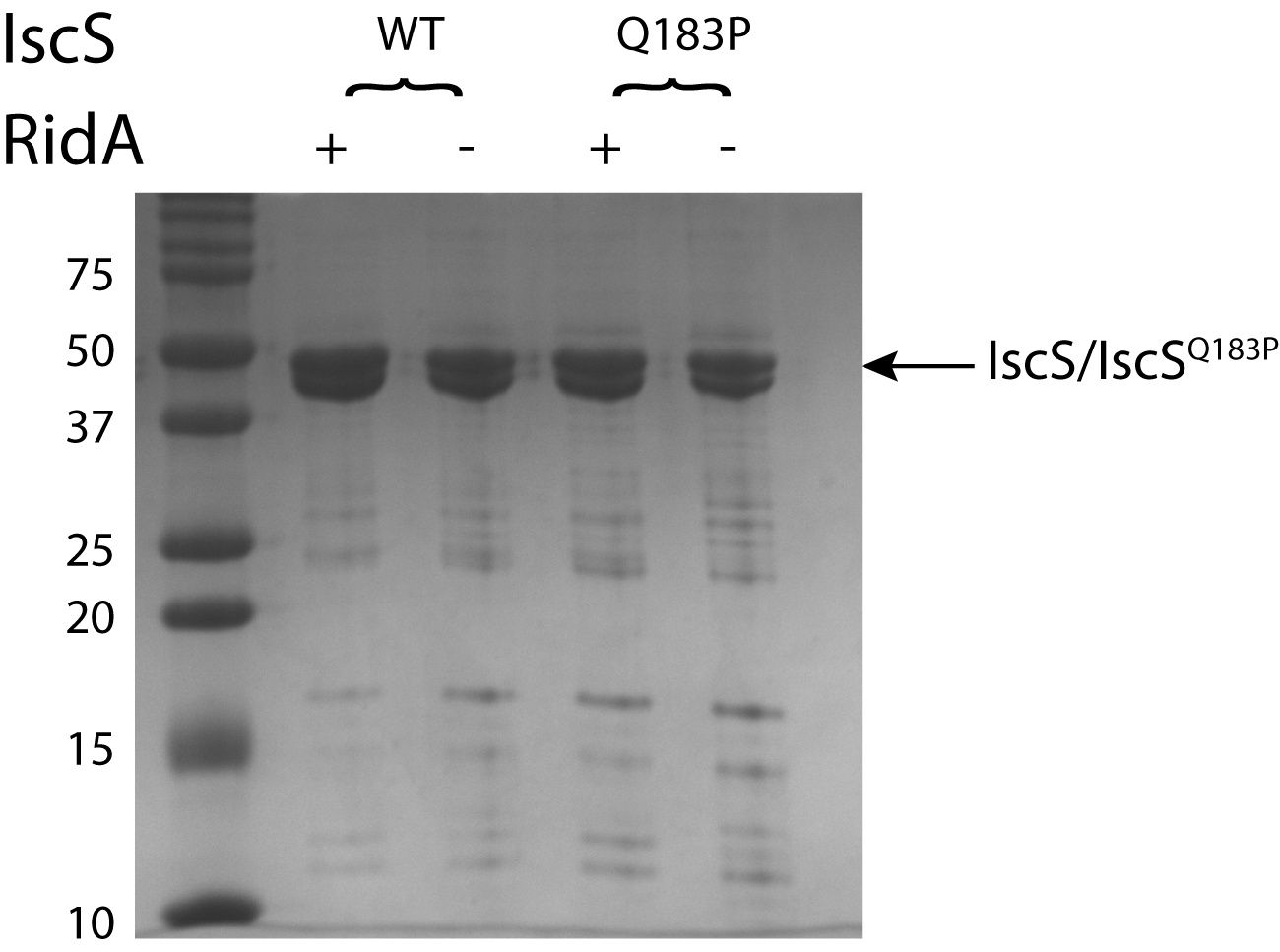

Supplement: FIG S1 [file mbio.01071-22-s0002.tif]
